# Shorter survival in adolescent and young adult patients, compared to adult patients, with stage IV colorectal cancer in Japan

**DOI:** 10.1186/s12885-018-4241-9

**Published:** 2018-03-27

**Authors:** Dai Shida, Yuka Ahiko, Taro Tanabe, Takefumi Yoshida, Shunsuke Tsukamoto, Hiroki Ochiai, Atsuo Takashima, Narikazu Boku, Yukihide Kanemitsu

**Affiliations:** 1 Colorectal Surgery Division, National Cancer Center Hospital, 5-1-1 Tsukiji, Chuo-ku, Tokyo, 1040045 Japan; 2 Gastrointestinal Medical Oncology Division, National Cancer Center Hospital, 5-1-1 Tsukiji, Chuo-ku, Tokyo, 1040045 Japan

**Keywords:** Adolescent and young adult, Stage IV, Colorectal cancer (CRC)

## Abstract

**Background:**

The incidence of colorectal cancer in adolescent and young adult patients is increasing. However, survival and clinical features of young patients, especially those with stage IV disease, relative to adult patients remain unclear.

**Methods:**

This retrospective single-institution cohort study was conducted at a tertiary care cancer center. Subjects were 861 consecutive patients who were diagnosed with stage IV colorectal cancer at the age of 15 to 74 years and who were referred to the division of surgery or gastrointestinal oncology at the National Cancer Center Hospital from 1999 to 2013. Overall survival (OS) was investigated and clinicopathological variables were analyzed for prognostic significance.

**Results:**

Of these, 66 (8%) were adolescent and young adult patients and 795 (92%) were adult patients. Median survival time was 13.6 months in adolescent and young adult patients and 22.4 months in adult patients, and 5-year OS rates were 17.3% and 20.3%, respectively, indicating significant worse prognosis of adolescent and young adult patients (*p* = 0.042). However, age itself was not an independent factor associated with prognosis by multivariate analysis. When compared with adult patients, adolescent and young adult patients consisted of higher proportion of the patients who did not undergo resection of primary tumor, which was an independent factor associated with poor prognosis in multivariate analysis. In patients who did not undergo resection (*n* = 349), OS of adolescent and young adult patients were significantly worse (*p* = 0.033).

**Conclusions:**

Prognoses were worse in adolescent and young adult patients with stage IV colorectal cancer compared to adult patients in Japan, due to a higher proportion of patients who did not undergo resection with more advanced and severe disease, but not due to age itself.

## Background

The incidence of colorectal cancer (CRC) in adolescent and young adult patients has been increasing steadily over the past two to three decades, although the absolute number of patients remains relatively low. [1, 2] A recent study based on the Surveillance, Epidemiology, and End Results database (from 1975 to 2010) in the United States reported that the most pronounced increase in CRC incidence was observed in patients aged 20 to 34 years, with an annual percentage change of 1.99. [3] In Japan, CRC is the leading cancer among males and the fourth most common cancer among females following breast, cervix uteri, and thyroid cancers in the adolescent and young adult population (generally includes patients aged 15 to 39 years [4]), according to Cancer Statistics in Japan in 2016 (from 2008 to 2012). [5]

Adolescent and young adult patients with cancer generally tend to present at advanced stages, exhibiting aggressive tumor features compared to older patients. [6] Although CRC shows similar trend, few studies have focused specifically on CRC in young patients because of its low incidence and a unique age range straddling both “pediatric” and “adult” cohorts. CRC in young patients exhibits specific molecular and clinical characteristics associated with a distinct biological phenotype compared with older patients, with a greater frequency of mucinous histology and deficient mismatch repair. [7, 8] However, the clinical outcomes of young patients with CRC are not well-known. Previous reports have suggested that younger patients with CRC suffer from more aggressive disease and show worse prognoses than older adults. [9–11] However, when matched by tumor stage (stage I - III), survival rates appear to be similar—if not better—in young adults compared with older adults. [12, 13] On the contrary, a large population-based study of stage IV patients reported a 5-year stage-specific survival of 18.1% in younger patients (aged 20 to 40 years) compared to 6.2% in older patients (aged 60 to 80 years) based on data from the Surveillance, Epidemiology, and End Results database (from 1991 to 1999). [14] However, other than this report, few detailed data are available for young patients with stage IV CRC compared to adult patients, especially outside the United States. Thus, the idea that young patients with CRC have poorer outcomes compared to adult patients remains controversial, especially for stage IV CRC.

The present study aimed to evaluate the prognoses of adolescent and young adult patients with stage IV CRC compared to adult patients in terms of overall survival (OS). Patients were collected from both surgery and gastrointestinal oncology divisions of the National Cancer Center Hospital, thereby allowing us to investigate the entire patient cohort with a diagnosis of stage IV CRC, including non-resected patients. We also explored the difference in clinical or pathological factors by age associated with differences in survival.

## Methods

### Study population

Subjects were 861 consecutive patients who were diagnosed with stage IV CRC at the age of 15 to 74 years, and who were referred to the surgery or gastrointestinal oncology division of the National Cancer Center Hospital between January 1997 and December 2013. Patients who were initially diagnosed to have stage IV CRC with histologic diagnoses of adenocarcinoma were included. Other histological types were excluded. Since our analysis focused on a comparison of adolescent and young adult patients (aged 15–39 years) [4] with adult patients (aged 40–74 years), we excluded patients aged ≥75 or < 15 years. The reason why excluded the aged patients (≥75 years) is that, because the evidence of the safety and effectiveness of oxaliplatin-based chemotherapy is lacking in the Asian population, systemic chemotherapy without oxaliplatin are recognized as standard treatments in Japan, which is clearly different from that for young and adult patients.

This retrospective study was approved by the Institutional Review Board (IRB) of the National Cancer Center Hospital (IRB code: 2015–320).

### Subgroup analysis

Among stage IV CRC, prognosis of patients who underwent curative resection differed from that of patients with unresectable stage IV CRC. Moreover, among unresectable stage IV CRC, palliative resection of the primary tumor may be associated with improved OS as we reported previously. [15] Thus, the subjects were classified into the following subgroups: patients who underwent primary tumor resection with metastasectomy (i.e., curative resection), patients who underwent primary tumor resection without metastasectomy (i.e., palliative resection of the primary tumor), and patients who did not undergo resection. Curative resection included R0 resection for peritoneal metastasis as we reported previously. [16]

### Data collection

The following parameters were assessed in the medical records retrospectively: age, treatment year, gender, Eastern cooperative oncology group performance status (PS), symptoms (asymptomatic/symptomatic), location of primary tumor (proximal colon including the cecum, hepatic flexure, and transverse colon; distal colon including the splenic flexure, sigmoid, and rectosigmoid junction; and rectum), histological type, and serum carcinoembryonic antigen (CEA) levels before treatment. The M category was assessed according to the Union for International Cancer Control TNM classification (8th edition), which was recently revised to include the following three subcategories: M1a (metastasis to one organ, excluding peritoneum), M1b (metastasis to more than one organ, excluding peritoneum), and M1c (metastasis to the peritoneum, with or without other organ involvement). [17] The grade of liver metastasis was also assessed according to the Japanese classification of colorectal carcinoma by the Japan Society for Cancer of the Colon and Rectum [18]. In this Japanese classification, grade of liver metastasis is determined according to the number and the maximum diameter of the metastatic lesion(s), and H3 is defined as diffuse liver metastases (≥ 5 lesions) with the maximum diameter of hepatic metastases > 5 cm.

### Follow-up

Follow-up consisted of serum tumor marker measurements every one to three months and computed tomography (CT) every three to six months, with cut-off date of July 2017. Complete follow-up was conducted for the entire cohort of patients, with a median follow-up time for survivors of 47 months (range, 2–144 months).

### Statistical analysis

Pearson’s chi-square test for categorical variables and the Wilcoxon rank-sum test for continuous variables were used to compare various factors in both groups. OS was defined as the interval between the date of stage IV CRC diagnosis and the date of death for all-cause, and censored for survivors at the date of the data cut-off. The Kaplan-Meier method was used to estimate OS. Differences in survival outcomes were assessed by the log-rank test. Multivariate Cox proportional hazards regression models were performed to evaluate the prognostic impact of age (adolescent and young adult or adult) with OS, adjusting for key clinicopathological factors (treatment year, PS, symptoms, primary tumor location, M category, grade of liver metastasis, tumor differentiation, preoperative CEA levels, and subgroups classified by type of resection). Results are presented as hazard ratios (HRs) and 95% confidence intervals (CIs).

Data are expressed as numbers of patients, ratios (%), or HRs and 95% CIs, as indicated. *P* < 0.05 was considered statistically significant. All statistical analyses were performed using the JMP12 software program (SAS Institute Japan Ltd., Tokyo, Japan).

## Results

### Characteristics of the study cohort

**Fig. 1:**
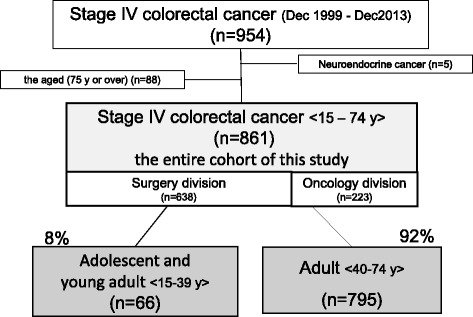
Characteristics of the study cohort of the 954 patients with stage IV colorectal cancer initially collected, those aged ≥75 years (*n* = 88), or those who had neuroendocrine cancer (*n* = 5), were excluded, leaving a total of 861 patients who were subjected to analysis. Of these, 638 had been referred to the surgery division and 223 to the gastrointestinal oncology division

Figure 1 shows the characteristics of the study cohort. Between December 1999 and December 2013, a total of 954 patients with stage IV CRC were referred to the National Cancer Center. [15] Of these, patients with histologic diagnoses other than adenocarcinoma (e.g., neuroendocrine tumor) (*n* = 5) and eighty-eight patients aged ≥75 years were also excluded. Ultimately, 861 patients met the aforementioned inclusion criteria, of whom 638 had been referred to the surgery division and 223 to the gastrointestinal medical oncology division.

Sixty six (8%) were adolescent and young adult patients and 795 (92%) were adult patients. Table 1 summarizes the characteristics of adolescent and young adult and adult patients. Treatment year, gender, and PS ratios did not differ between the two groups (*p* = 0.291, *p* = 0.094 and *p* = 0.841, respectively). Significant group-dependent differences were observed in symptoms (asymptomatic/symptomatic) (*p* = 0.026). Tumor location (*p* = 0.663), distribution of M subcategory (*p* = 0.784), tumor differentiation (*p* = 0.217), and preoperative CEA levels (*p* = 0.672) did not differ between the two groups.

**Table 1:** Clinical characteristics of patients

Variable	Category		Entire cohort (*n* = 861)				
			Adolescent and young adult patients *n* = 66		Adult patients *n* = 795		*p* value
Treatment Year	1999–2004		25 (38%)		251 (32%)		0.291
	2005–2013		41 (62%)		544 (68%)		
Gender	Male		31 (47%)		458 (58%)		0.094
	Female		35 (53%)		337 (42%)		
ECOG performance status	PS 0, PS1		56 (93%)		702 (94%)		0.841
	PS2, PS3, PS4		4 (7%)		45 (6%)		
	missing data		6		48		
Symptoms	Asymptomatic		9 (14%)		207 (26%)		0.026
	Symptomatic		56 (86%)		588 (74%)		
Tumor location	Proximal colon		21 (32%)		227 (29%)		
	Distal colon		26 (39%)		359 (45%)		0.663
	Rectum		19 (29%)		209 (26%)		
M category	M1a		34 (52%)		404 (51%)		
	M1b		14 (21%)		196 (25%)		0.784
	M1c		18 (27%)		195 (24%)		
Tumor differentiation	Differentiated		57 (86%)		692 (87%)		
	Poorly differentiated		6 (9%)		76 (10%)		0.217
	Mucinous		1 (2%)		20 (2%)		
	Others		2 (3%)		7 (1%)		
Preoperative CEA levels	< 30 ng/ml		35 (53%)		399 (50%)		0.672
	≥30 ng/ml		31 (47%)		394 (50%)		
Primary tumor resection	Yes	Curative resection	13 (20%)	34 (52%)	210 (26%)	508 (64%)	0.029
		Metastasectomy (−)	21 (32%)		298 (37%)		
	No	chemotherapy	30 (45%)	32 (48%)	265 (33%)	287 (36%)	
		BSC	2 (3%)		22 (3%)		

Data are presented as n (%)

*CEA*: carcinoembryonic antigen, *BSC*: best supportive care, *ECOG*: eastern cooperative oncology group

Decisions about initial treatment were typically made by colorectal surgeons, medical oncologists, hepatobiliary surgeons, and radiologist (multidisciplinary team), who took into account disease severity and patient condition including comorbidities. Of all stage IV patients, 223 (26%) underwent resection of both the primary tumor and tumors at metastatic sites as curative resection, including 13 adolescent and young adult patients (20% of all adolescent and young adult patients) and 210 adult patients (26% of all adult patients). Resected metastatic sites in these 223 patients included the liver (*n* = 148), peritoneum (*n* = 27), lung (*n* = 16), and other or several sites (*n* = 32). Of the remaining 638 stage IV patients with unresectable tumors, 319 (50%) underwent palliative resection of the primary tumor, and 319 (50%) did not. Patients who underwent diverting stoma construction without resection of the primary tumor (*n* = 77), bypass surgery (*n* = 13), or probe laparotomy (*n* = 8) were included in the unresected group. In the palliative resection group, 41 patients were treated with only 5-fluorouracil, 143 patients with combination of cytotoxic agents (fluorouracil plus oxaliplatin and/or irinotecan), and 99 patients with at least one targeted agents (bevacizumab and/or anti- epidermal growth factor receptor antibody), and 2 patients with best supportive care (and missing data of 34 patients). In the unresected group, 46 patients were treated with only 5-fluorouracil, 152 patients with combination of cytotoxic agents, 94 patients with at least one targeted agents, and 22 patients with best supportive care (and missing data of 5 patients). The ratios of primary tumor resection also differed significantly between the two groups (*p* = 0.029) (Table 1). Specifically, adolescent and young adult patients tended not to undergo primary tumor resection, including palliative resection.

### Long-term outcomes of patients with stage IV CRC

**Fig. 2:**
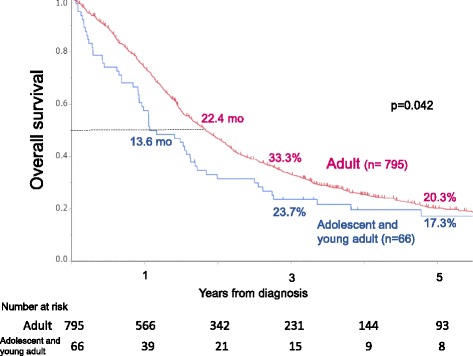
(*upper left*) Overall survival curves of adolescent and young adult (15–39 y) and adult (40–74 y) patients with stage IV colorectal cancer. Of the entire cohort (*n* = 985), 66 (8%) were adolescent and young adult patients and 795 (92%) were adult patients

Median survival time was 13.6 months in adolescent and young adult patients and 22.4 months in adult patients, associated with the 3-year OS rates of 23.7% in adolescent and young adult patients and 33.3% in adult patients and the 5-year OS rates of 17.3% and 20.3%, respectively (*p* = 0.042) (Fig. 2).

**Fig. 3:**
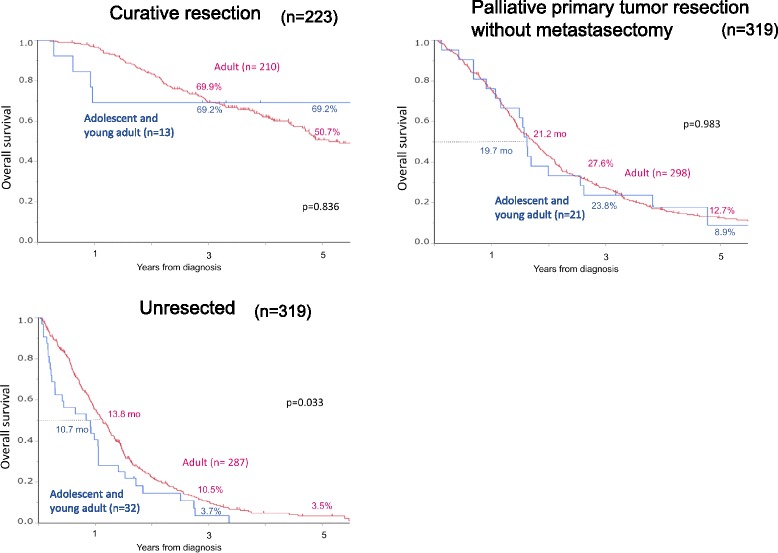
Overall survival of adolescent and young adult and adult patients with stage IV colorectal cancer in three subgroups: patients who underwent curative resection (*n* = 223) (*upper right*), patients who underwent palliative primary tumor resection without metastasectomy (*n* = 319) (*upper right*), and patients who did not undergo resection (*n* = 319) (*lower left*)

Subgroup analysis was also examined by the following subgroups: patients who underwent curative resection (*n* = 223), patients who underwent primary tumor resection without metastasectomy (*n* = 319), and patients who did not undergo resection (n = 319). Figure 3 shows survival curves of young and adult patients with stage IV CRC in three subgroups. In patients who did not undergo resection (*n* = 349), median survival time was 10.7 months in young patients and 13.8 months in adult patients, associated with the 3-year OS rates of 3.7% in young patients and 10.5% in adult patients and the 5-year OS rates of 0% and 3.5%, respectively (*p* = 0.033). In contrast, prognoses of young patients and of adult patients did not differ in curative resection patients (*p* = 0.836) and in palliative resection patients (*p* = 0.983).

### Factors affecting the prognosis of patients with stage IV CRC

In univariate analysis, age was associated with prognosis (*p* = 0.042), and treatment year (*p* < 0.0001), PS (*p* < 0.0001), symptoms (*p* = 0.039), tumor location (*p* < 0.0001), M category (*p* < 0.0001), grade of liver metastasis (*p* < 0.0001), tumor differentiation (*p* < 0.0001), preoperative CEA levels (*p* < 0.0001), and primary tumor resection (*p* < 0.0001) were also significantly associated with OS (Table 2). Multivariate Cox proportional hazards regression models revealed that treatment year (1999–2004), tumor location (proximal colon), and widespread liver metastasis were independent factors associated with a worse prognosis (Table 2), whereas PS (PS 0–1), M1a subcategory, differentiated adenocarcinoma, preoperative CEA level ≤ 30 ng/ml, and curative resection were independent factors associated with better prognosis (Table 2). On the other hand, age (young versus adult) was not a significant prognostic factor.

**Table 2:** Univariate and multivariate analyses of factors affecting survival in stage IV colorectal cancer patients

Variable	Category		n	Median overall survival (months)	Univariate analysis *p* value	Multivariate analysis		
						Hazard ratio	95% CI	*p* value
Age	Adolescent and young adult (15–39 y)		66	13.6 (11.2–19.8)	0.042	Reference		
	Adult (40–74 y)		795	22.4 (20.2–24.8)		0.88	0.66–1.21	0.423
Treatment Year	1999–2004		276	15.1 (13.6–17.3)	< 0.0001	Reference		
	2005–2013		585	25.7 (23.2–29.0)		0.64	0.53–0.76	< 0.0001
Gender	Male		489	22.8 (20.2–25.2)	0.747			
	Female		372	19.9 (18.1–23.5)				
Performance status	PS 0, PS1		758	24.3 (22.1–26.5)	< 0.0001	Reference		
	PS2, PS3, PS4		49	16.4 (6.0–7.6)		3.81	2.70–5.25	< 0.0001
Symptoms	Asymptomatic		216	26.4 (22.5–32.0)	0.039	1		
	Symptomatic		644	20.1 (18.2–22.2)		1.13	0.94–1.37	0.185
Tumor location	Proximal colon		248	16.4 (13.5–18.0)	< 0.0001	Reference		
	Distal colon		385	24.2 (21.5–26.9)		0.67	0.55–0.81	< 0.0001
	Rectum		228	26.4 (21.3–32.0)		0.75	0.60–0.93	0.010
M category	M1a		438	32.0 (26.9–35.7)	< 0.0001	Reference		
	M1b		210	17.4 (15.2–19.6)		1.31	1.05–1.62	0.015
	M1c		213	16.2 (13.7–18.5)		1.46	1.18–1.79	0.001
Liver metastasis	None or not severe		298	35.0 (29.1–42.7)	< 0.0001	Reference		
	Widespread (H3)		360	15.3 (13.9–16.8)		1.67	1.37–2.04	< 0.0001
Tumor differentiation	Differentiated		749	23.9 (21.5–26.0)	< 0.0001	Reference		
	Poorly differentiated		82	12.4 (7.8–13.9)		1.84	1.38–2.39	< 0.0001
	Mucinous		21	15.6 (8.0–25.1)		1.52	0.83–2.56	0.167
	Others		9	–		–	–	–
Preoperative CEA levels	< 30 ng/ml		434	27.6 (23.4–32.1)	< 0.0001	Reference		
	≥30 ng/ml		424	17.5 (15.9–19.1)		1.08	0.90–1.29	0.420
Primary tumor resection	Yes	Curative resection	223	68.0 (57.1–110.6)	< 0.0001	Reference		
		Metastasectomy(−)	319	20.1 (18.4–23.4)		2.52	1.95–3.28	< 0.0001
	No		319	13.1 (11.8–15.0)		4.15	3.14–5.52	< 0.0001

Data are presented as median (95%CI) or hazard ratios (95%CIs)

*CI*: confidence interval, *CEA*: carcinoembryonic antigen

*H3*: diffuse liver metastases (≥ 5 lesions) with the maximum diameter of hepatic metastases > 5 cm, as defined according to the Japanese classification of colorectal carcinoma, the Japan Society for Cancer of the Colon and Rectum

Taken these results together, prognoses were worse in adolescent and young adult patients with stage IV colorectal cancer compared to adult patients, due to a higher proportion of patients who did not undergo resection with more advanced and severe disease, but not due to age itself.

## Discussion

The subjects of the present study are all stage IV patients who were referred to the divisions of surgery and gastrointestinal oncology at the National Cancer Center Hospital, including patients who did not undergo resection (319 patients; 37% of the entire cohort). The median survival time was 13.6 months in adolescent and young adult patients, with 3- and 5-year OS rates of 23.7% and 17.3%, respectively, which resulted in significant worse prognosis compared to adult patients (*p* = 0.042). Subgroup analysis reveals that in patients who did not undergo resection, OS of young patients were significantly worse (*p* = 0.033), whereas prognoses did not differ in curative resection patients and in palliative resection patients. Among patients who did not undergo resection, young patients consisted of the higher proportion of the patients with bone metastasis, which portends a poor prognosis [19], when compared with the adult patients (13% vs 9%) (data not shown). Similarly, young patients consisted of the higher proportion of the patients who had peritoneal metastasis (M1c) with other distant metastasis, which seems to be poorest prognosis among stage IV [20], compared to the adult patients (22% vs 18%) (data not shown). These results may account for why young patients are less likely to have resection, and why prognosis of young patients is worse. Taken together with multivariate analysis, the worse prognosis observed in young patients is likely due to a higher proportion of patients who did not undergo resection with more advanced and severe disease.

A recent study conducted in the United States reported that survival after a CRC diagnosis at a young age is significantly worse in non-Hispanic black patients with stage IV CRC compared with non-Hispanic white patients, based on the Surveillance, Epidemiology, and End Results data. [21] Another study reported poorer OS in African American patients with stage IV CRC compared with European American patients, especially among those aged ≤50 years. [22] These reports suggest that racial differences affect survival in younger patients with stage IV CRC. At present, no detailed outcome data are available for other races, including Asians. Since all subjects of our study were Asians, including three non-Japanese patients, our results represent survival data for Asian patients with stage IV CRC. The 5-year OS was 17% of adolescent and young adult patients in this study, which is very similar to the 5-year OS reported in the United States for young patients (≤40 years) with stage IV CRC. [23]

There are some potential limitations to this study. First, since the present study was retrospective in design, there may be biases related to surgical treatment and chemotherapy regimens. Second, although consecutive patients were enrolled, there have been significant changes during the long study period (1999 to 2013) in treatment strategies, such as chemotherapy, as well as perioperative awareness regarding peritoneal metastasis. Third, our study lacked mismatch repair data which might affect the prognosis, although deficient mismatch repair seems rare in stage IV patients with CRC and the majority of patients with stage IV CRC are sporadic cases, rather than familial. [24] Nonetheless, our observations warrant further consideration and validation in a larger patient series of young patients with stage IV CRC.

In conclusion, adolescent and young adult patients with stage IV CRC has worse prognoses compared to adult patients in Japan. The worse prognosis observed in young patients is likely due to a higher proportion of patients who did not undergo resection with more advanced and severe disease. The question why young people have more aggressive disease needs answering by future studies.

## Abbreviations

CEA: Carcinoembryonic antigen
CRC: Colorectal cancer
CT: Computed tomography
OS: Overall survival
